# ﻿Three new species of *Fomitiporia* (Hymenochaetales, Basidiomycota) from tropical dry forests in Ecuador and Peru

**DOI:** 10.3897/mycokeys.118.154175

**Published:** 2025-06-10

**Authors:** Jian Chen, Josef Vlasák, Kai-Yue Luo, Yuan Yuan

**Affiliations:** 1 State Key Laboratory of Efficient Production of Forest Resources, School of Ecology and Nature Conservation, Beijing Forestry University, Beijing 100083, China Beijing Forestry University Beijing China; 2 Biology Centre of the Academy of Sciences of the Czech Republic, České Budějovice, Czech Republic Biology Centre of the Academy of Sciences of the Czech Republic České Budějovice Czech Republic

**Keywords:** Hymenochaetaceae, phylogeny, taxonomy, wood-decaying fungi

## Abstract

Three new species in Hymenochaetaceae are illustrated and described from tropical Ecuador and Peru based on morphology and phylogenetic analyses. The species *Fomitiporiarobustiformis* is characterized by perennial, pileate basidiocarps; ungulate pilei; brown and glabrous pileal surface; broad and rounded margin; yellowish-brown pore surface; circular to angular pores measuring 6–7 per mm; thick and entire dissepiments; a dimitic hyphal system; the presence of fusoid cystidioles; the presence of hymenial setae; and globose basidiospores measuring 5–6.4 × 4.7–6.1 µm. *Fomitiporiaroseo-bubalina* is characterized by annual to perennial, resupinate to pileate basidiocarps; ungulate to triquetrous pilei; grayish and glabrous pileal surface; blunt margin; pale gray and glossy pore surface; circular to angular pores measuring 4–5 per mm; thick and entire dissepiments; a dimitic hyphal system; the presence of hymenial setae and cystidioles; and globose basidiospores measuring 5.3–6.7 × 5–6.2 µm. *Fomitiporiatriqueter* is characterized by perennial, pileate, sessile basidiocarps; triquetrous pilei with sharp margins; grayish dark and cracked pileal surfaces with concentrically sulcate, pale yellowish brown pore surfaces; circular pores measuring 9–10 per mm; the absence of hymenial setae and cystidioles; the presence of large rhomboid crystals in the hymenium; and globose basidiospores measuring 3.4–5.5 × 3.2–5.4 µm. The differences between the new species and morphologically similar and phylogenetically related species are also discussed.

## ﻿Introduction

Hymenochaetales is a major group of wood-inhabiting fungi, and more than 1,500 species have been reported in the order (Du et al. 2020; Wu et al. 2022a; Wang et al. 2023; Zhou et al. 2023; Dong et al. 2024; Zhao et al. 2024a, 2024b; Liu et al. 2025; Viner 2025; Yang et al. 2025). Hymenochaetaceae is the main family of the order, in which Wu et al. (2022a) made extensive and systematic studies on the family. *Fomitiporia* Murrill (Murrill 1907), as a genus of Hymenochaetaceae typified by *F.langloisii* Murrill, is a worldwide and important genus (Wu et al. 2022b). The genus is rich in species diversity, and many new species were recently described from subtropical and tropical areas (Dai et al. 2021). The largest fungus was found in the genus (Dai and Cui 2011). Some species of the genus are forest pathogens, such as *F.capensis* M. Fisch., M. Cloete, L. Mostert & F. Halleen, *F.hippophaëicola* (H. Jahn) Fiasson & Niemelä, *F.pseudopunctata* (A. David et al.) Fiasson (=*F.mediterranea* M. Fisch.), and *F.torreyae* Y.C. Dai & B.K. Cui (Fischer 2002; Dai et al. 2007; Cloete et al. 2014; Yuan et al. 2023); and some species have medicinal values, such as *F.ellipsoidea* B.K. Cui & Y.C. Dai, *F.hartigii* (Allesch. & Schnabl) Fiasson & Niemelä (≡ *Phellinushartigii* (Allesch. & Schnabl) Pat.), *F.punctata* (P. Karst.) Murrill, and *F.robusta* (P. Karst.) Fiasson & Niemelä (≡ *Phellinusrobustus* (P. Karst.) Bourdot & Galzin) (Dai et al. 2009; Wu et al. 2019; Dai 2022, 2023; Zhang et al. 2023, 2024; Ghobad-Nejhad et al. 2024; Shen et al. 2024). In traditional *Phellinuss.l.* (Fischer 1996), *Fomitiporia* has been called the ‘*Phellinusrobustus* complex,’ and it was confirmed as a homogeneous genus in Hymenochaetaceae based on molecular data (Wagner and Fischer 2001). The genus is characterized by mostly perennial, pileate or resupinate basidiocarps, homogeneous context, a dimitic hyphal system, hymenial setae rarely present, basidiospores broadly ellipsoid, subglobose or globose, hyaline, thick-walled, smooth, mostly dextrinoid and cyanophilous; on angiosperms and gymnosperms, and causing a white rot (Chen and Cui 2017; Chen et al. 2021; Wu et al. 2022b).

Numerous studies have dealt with *Fomitiporia* in the last 20 years, and species in *Fomitiporia* have been documented to have a higher than presumed phylogenetic and taxonomic diversity (Vlasák and Vlasák 2016; Liu et al. 2018). Because of the similarity in morphology, many new species of *Fomitiporia* have been distinguished by their phylogenetic and geographical characteristics (Chen and Cui 2017). Recently, based on molecular and morphological analyses, more new species were described from the tropics, such as *F.tasmanica* G.M. Gates, X.H. Ji & Jia J. Chen, *F.eucalypti* Y.C. Dai & X.H. Ji, and *F.rondonii* Alves-Silva & Drechsler-Santos (Chen et al. 2021; Wu et al. 2022b; Manawasinghe et al. 2024). About 70 species of *Fomitiporia* are currently reported worldwide (Wu et al. 2022b).

During a study of wood-decaying fungi in South America, three undescribed species matching the concept of *Fomitiporia* were discovered. To understand their taxonomic placement, phylogenetic analysis was carried out based on the internal transcribed spacers (ITS) and the nuclear large subunit ribosomal DNA (nLSU) sequences according to Wu et al. (2022b). Their morphological characteristics were also described and illustrated.

## ﻿Materials and methods

### ﻿Morphological studies

The studied specimens were collected from tropical dry forests in Ecuador and Peru (see Results for details of collection and field photographs). All vouchers are deposited in the Fungarium of the Institute of Microbiology, Beijing Forestry University (BJFC), Beijing, China. Morphological characterization (color, texture, taste, and odor of basidiocarps) was conducted based on field notes and voucher specimens. Microscopic analysis follows Chen et al. (2015), and the special color terms follow Petersen (1996). Mushroom sections were examined for microstructures using a Nikon Eclipse 80i compound microscope equipped with phase contrast optics, with magnification parameters ranging from 100× to 1000×. For measurements and drawings, slide preparations were stained with cotton blue, Melzer’s reagent, and 5% potassium hydroxide. Basidiospores were measured from sections cut from the tubes, and 5% of measurements were excluded from each end of the range and are given in parentheses. Abbreviations employed in descriptions: KOH, 5% aqueous potassium hydroxide solution; IKI, Melzer’s reagent (IKI[+]: dextrinoid; IKI-: both inamyloid and non-dextrinoid); CB, Cotton Blue affinity (CB+: cyanophilous; CB-: weakly cyanophilous); L, arithmetic average of spore length; W, arithmetic average of spore width; Q, L/W ratios; n, number of basidiospores/measured from given number of specimens.

### ﻿DNA extraction, amplification, and sequencing

The CTAB-based rapid plant genome extraction kit (Aidlab Biotechnologies Co., Ltd., Beijing) was used to obtain genomic DNA from dried specimens. The primer pair ITS4 and ITS5 was used for amplification of the ITS region (White et al. 1990), while the primer pair LR0R and LR7 (http://www.biology.duke.edu/fungi/mycolab/primers.htm) was used to amplify the nLSU region. The PCR procedure for ITS was as follows: initial denaturation at 95 °C for 3 min, followed by 35 cycles at 94 °C for 40 s, 54 °C for 45 s, and 72 °C for 1 min, and a final extension of 72 °C for 10 min. The PCR procedure for nLSU was as follows: initial denaturation at 94 °C for 1 min, followed by 35 cycles at 94 °C for 30 s, 50 °C for 1 min, and 72 °C for 1.5 min, and a final extension of 72 °C for 10 min. The PCR products were purified and sequenced at the Beijing Genomics Institute in China, using the same primers. The newly generated sequences were deposited in the GenBank database.

### ﻿Phylogenetic analyses

Reference ITS and nLSU sequences from various *Fomitiporia* species were downloaded from GenBank according to Wu et al. (2022b). Sequences of *Neophellinusuncisetus* (Robledo, Urcelay & Rajchenb.) Y.C. Dai, F. Wu, L.W. Zhou, Vlasák & B.K. Cui, obtained from GenBank, were used as the outgroup. All sequences analyzed in this study are listed in Table 1. ITS and nLSU sequences were aligned with BioEdit (Hall 1999) and ClustalX (Thompson et al. 1997). Then two datasets were separately aligned using MAFFT 7.110 (Katoh and Standley 2013) under the G-INS-i option. Ambiguous regions at the start and the end were deleted. Then, alignments were spliced and transformed into formats in Mesquite 3.2 (Maddison and Maddison 2017) and deposited in TreeBASE (http://www.treebase.org; accession number 32101; reviewer access URL: http://purl.org/phylo/treebase/phylows/study/TB2:S32101?x-access-code=f1ab253962f03e6e486b3b5ce8a04991&format=html). The phylogenetic analysis was performed using maximum likelihood (ML) and Bayesian inference (BI) methods based on ITS and nLSU datasets (Wu et al. 2022b). The best evolutionary model was estimated using jModelTest (Guindon and Gascuel 2003; Posada 2008) under the Akaike information criterion. ML and BI analyses were conducted using RAxML-HPC2 through the CIPRES Science Gateway, with all parameters set to their default settings (www.phylo.org; Miller et al. 2010). The BI analysis was conducted in two independent runs, each with four chains and starting from random trees. Trees were sampled every 100 generations from a total of 6 million generations. The first 25% of trees were removed, and the remaining ones were used to construct a 50% majority consensus tree and calculate Bayesian posterior probabilities (BPPs). All trees were viewed in FigTree 1.4.3. The two methods constructed nearly congruent topologies for each alignment. Therefore, only the topologies inferred from the ML method were presented along with bootstrap values from the ML method (≥ 50%) and BPPs (≥ 0.9) from the BI methods at the nodes.

**Table 1.** Information for the sequences used in this study.

Species name	Sample no.	Location	GenBank accession no.		References
			ITS	nLSU	
*Fomitiporiaaethiopica*	MUCL 44777	Ethiopia	GU478341	AY618204	Amalfi et al. (2010)
*Fomitiporiaaethiopica*	MUCL 44806	Ethiopia	GU461944	AY618202	Amalfi et al. (2010)
*Fomitiporiaalpina*	Dai 15735	China	KX639627	KX639645	Wu et al. (2022b)
*Fomitiporiaapiahyna*	MUCL 51485	Ecuador	GU461962	GU461996	Amalfi et al. (2010)
*Fomitiporiaatlantica*	FLOR 47591	Brazil	KU557529	—	Li et al. (2016)
*Fomitiporiaatlantica*	FLOR 58554	Brazil	KU557528	KU557526	Li et al. (2016)
*Fomitiporiaaustraliensis*	MUCL 49406	Australia	AY624997	GU462001	Chen et al. (2021)
*Fomitiporiabaccharidis*	MUCL 47756	Argentina	JQ087886	JQ087913	Wu et al. (2022b)
*Fomitiporiabaccharidis*	MUCL 47757	Argentina	JQ087887	JQ087914	Wu et al. (2022b)
*Fomitiporiabakeri*	MUCL 51098	USA	JQ087874	JQ087901	Wu et al. (2022b)
*Fomitiporiabambusarum*	ICN 200563	Brazil	MN918544	MN918537	Alves-Silva et al. (2020a)
*Fomitiporiabambusarum*	ICN 200564	Brazil	MN918545	MN918538	Alves-Silva et al. (2020a)
*Fomitiporiabambusipileata*	ICN 200559	Brazil	MN918546	MN918539	Alves-Silva et al. (2020a)
*Fomitiporiabambusipileata*	ICN 200557	Brazil	MN918548	MN918541	Alves-Silva et al. (2020a)
*Fomitiporiabannaensis*	MUCL 46926	Thailand	KF444682	KF444705	Wu et al. (2022b)
*Fomitiporiabannaensis*	MUCL 46930	China	KF444683	KF444706	Wu et al. (2022b)
*Fomitiporiacalkinsii*	MUCL 51099	USA	KF444686	KF444709	Wu et al. (2022b)
*Fomitiporiacalkinsii*	MUCL 51398	USA	KF444687	KF444710	Wu et al. (2022b)
*Fomitiporiacapensis*	MUCL 53009	South Africa	JQ087890	JQ087917	Wu et al. (2022b)
*Fomitiporiacapensis*	CMW 48613	South Africa	MH599112	MH599122	Wu et al. (2022b)
*Fomitiporiacarpinea*	Dai 18023	China	MH930812	MH930810	Wu et al. (2022b)
*Fomitiporiacastilloi*	MUCL FG10282	French Guiana	JQ087889	JQ087916	Wu et al. (2022b)
*Fomitiporiacastilloi*	MUCL 53980	French Guiana	JX093786	JX093830	Wu et al. (2022b)
*Fomitiporiachilensis*	BAFC 52942	Chile	NR_164591	NG_068862	Rajchenberg et al. (2019)
*Fomitiporiachilensis*	CIEFAPcc586	Chile	MK131090	MK193751	Rajchenberg et al. (2019)
*Fomitiporiachilensis*	CIEFAPcc587	Chile	MK131094	MK193752	Rajchenberg et al. (2019)
*Fomitiporiaconyana*	FLOR 58547	Brazil	KU663298	KU663270	Alves-Silva et al. (2020b)
*Fomitiporiaconyana*	FLOR 58548	Brazil	KU663299	KU663271	Alves-Silva et al. (2020b)
*Fomitiporiacupressicola*	MUCL 52489	Mexico	JQ087879	JQ087906	Amalfi et al. (2012)
*Fomitiporiacupressicola*	MUCL 52490	Mexico	JQ087880	JQ087907	Amalfi et al. (2012)
*Fomitiporiadeserticola*	JV 1209/40-J	USA	KT381634	—	Vlasák and Vlasák (2016)
*Fomitiporiadeserticola*	JV 1209/41-J	USA	KT381633	—	Vlasák and Vlasák (2016)
*Fomitiporiadeserticola*	JV 1209/46	USA	KT381632	—	Vlasák and Vlasák (2016)
*Fomitiporiadryophila*	MUCL 46380	USA	EF429238	EF429219	Wu et al. (2022b)
*Fomitiporiadryophila*	MUCL 51144	USA	KF444689	KF444712	Wu et al. (2022b)
*Fomitiporiaelegans*	FURB 44484	Brazil	KU663320	—	Alves-Silva et al. (2020b)
*Fomitiporiaelegans*	FLOR 58556	Brazil	KU663319	KU663293	Alves-Silva et al. (2020b)
*Fomitiporiaerecta*	MUCL 49871	France	GU461939	GU461976	Wu et al. (2022b)
*Fomitiporiaeucalypti*	Dai 18586A	Australia	MH971172	MH971217	Wu et al. (2022b)
*Fomitiporiaeucalypti*	Dai 18682	Australia	MH971171	MH971216	Wu et al. (2022b)
*Fomitiporiaexpansa*	MUCL 55026	French Guiana	KJ401031	KJ401032	Amalfi and Decock (2014)
*Fomitiporiafissurata*	JV 1305/4-J	USA	KT381629	—	Vlasák and Vlasák (2016)
*Fomitiporiafissurata*	JV 1305/5-J	USA	KT381630	—	Vlasák and Vlasák (2016)
*Fomitiporiafissurata*	JV 1307/5-J	USA	KT381631	—	Vlasák and Vlasák (2016)
*Fomitiporiagabonensis*	MUCL 47576	Gabon	GU461971	GU461990	Wu et al. (2022b)
*Fomitiporiagaoligongensis*	Cui 8261	China	KX639624	KX639642	Chen and Cui (2017)
*Fomitiporiagatesiae*	Dai 18680	Australia	MH971169	MH971214	Wu et al. (2022b)
*Fomitiporiagatesiae*	Dai 18681	Australia	MH971170	MH971215	Wu et al. (2022b)
*Fomitiporiahartigii*	MUCL 53551	Estonia	JX093789	JX093833	Wu et al. (2022b)
*Fomitiporiahesleri*	01-712/2	-	AY340031	—	Fischer and Binder (2004)
*Fomitiporiahesleri*	01-77/4	-	AY340026	—	Fischer and Binder (2004)
*Fomitiporiahippophaëicola*	MUCL 31746	Belgium	GU461945	AY618207	Amalfi et al. (2010)
*Fomitiporiahippophaëicola*	MUCL 31747	Belgium	GU461946	GU461977	Amalfi et al. (2010)
*Fomitiporiaignea*	TX15	USA	MN108101	MN113927	Brown et al. (2020)
*Fomitiporiaignea*	TX16	USA	MN108102	MN113928	Brown et al. (2020)
*Fomitiporiaivindoensis*	MUCL 51311	Gabon	GU461952	GU461979	Amalfi et al. (2010)
*Fomitiporiaivindoensis*	MUCL 51312	Gabon	GU461951	GU461978	Amalfi et al. (2010)
*Fomitiporialagerstroemiae*	Dai 18335	Vietnam	MH930813	MH930811	Wu et al. (2022b)
*Fomitiporialangloisii*	MUCL 46375	USA	EF429242	EF429225	Wu et al. (2022b)
*Fomitiporiamaxonii*	MUCL 46017	Cuba	EF433559	EF429230	Wu et al. (2022b)
*Fomitiporiamaxonii*	MUCL 46037	Cuba	EF433560	EF429231	Wu et al. (2022b)
*Fomitiporianubicola*	FLOR 57850	Brazil	KU663303	NG_073602	Alves-Silva et al. (2020b)
*Fomitiporianubicola*	FLOR 57857	Brazil	KU663309	KU663282	Alves-Silva et al. (2020b)
*Fomitiporianeotropica*	MUCL 53114	French Guiana	JX093792	JX093836	Wu et al. (2022b)
*Fomitiporianobilissima*	MUCL 47580	Gabon	GU461966	GU461985	Wu et al. (2022b)
*Fomitiporianobilissima*	MUCL 51289	Gabon	GU461965	GU461984	Wu et al. (2022b)
*Fomitiporianorbulingka*	Cui 9722	China	KU364419	KU364429	Chen et al. (2017)
*Fomitiporianorbulingka*	Cui 9770	China	KU364420	KU364430	Chen et al. (2017)
*Fomitiporianorbulingka*	Cui 9766	China	KU364417	KU364427	Chen et al. (2017)
*Fomitiporiaovoidospora*	Dai 18283	Vietnam	MH971167	MH971212	Wu et al. (2022b)
*Fomitiporiaovoidospora*	Dai 18349	Vietnam	MH971168	MH971213	Wu et al. (2022b)
*Fomitiporiapentaphylacis*	Yuan 6012	China	JQ003900	JQ003901	Zhou and Xue (2012)
*Fomitiporiapolymorpha*	MUCL 46166	USA	GU461955	DQ122393	Wu et al. (2022b)
*Fomitiporiapolymorpha*	MUCL 46167	USA	GU461956	EF429233	Wu et al. (2022b)
*Fomitiporiapseudopunctata*	MUCL 38514	France	GU461953	AY618201	Wu et al. (2022b)
*Fomitiporiapseudopunctata*	MUCL 45670	France	GU461954	GU461980	Wu et al. (2022b)
*Fomitiporiapseudopunctata*	MUCL 51325	Czech	GU461948	GU461981	Wu et al. (2022b)
*Fomitiporiapseudopunctata*	MUCL 46168	France	JQ087891	JQ087918	Wu et al. (2022b)
*Fomitiporiapunctata*	MUCL 47629	Japan	GU461950	GU461982	Amalfi et al. (2010)
*Fomitiporiapunctata*	Dai 15772	China	KX639629	KX639647	Wu et al. (2022b)
*Fomitiporiapunicata*	Cui 26	China	GU461975	GU461992	Wu et al. (2022b)
*Fomitiporiapunicata*	Dai 7175	China	KX639632	KX639650	Wu et al. (2022b)
*Fomitiporiapunicata*	Dai 10640	China	KX663825	KX639653	Wu et al. (2022b)
*Fomitiporiarhamnoides*	Dai 17369	China	MH234388	MH234391	Liu et al. (2018)
*Fomitiporiarhamnoides*	GL-2016	China	KT861405	KY697187	Liu et al. (2018)
*Fomitiporiarhamnoides*	Dai 18091	China	MH234389	MH234392	Liu et al. (2018)
*Fomitiporiarobusta*	MUCL 51297	Estonia	JQ087892	JQ087919	Wu et al. (2022b)
*Fomitiporiarobusta*	MUCL 51327	Czech	GU461949	GU461993	Wu et al. (2022b)
***Fomitiporiarobustiformis***	**JV 2402/8**	**Ecuador**	**PV368088**	**PV368094**	**Present study**
***Fomitiporiarobustiformis***	**JV 2501/5-J**	**Peru**	**PV389821**	—	**Present study**
***Fomitiporiaroseo-bubalina***	**JV 2402/1**	**Ecuador**	**PV368089**	**PV368095**	**Present study**
***Fomitiporiaroseo-bubalina***	**JV 2402/10A**	**Ecuador**	**PV389820**	**PV389822**	**Present study**
*Fomitiporiasubtilissima*	FURB 47557	Brazil	NR_148098	NG_060426	GenBank
*Fomitiporiasubtilissima*	FURB 47437	Brazil	KU557530	—	GenBank
*Fomitiporiasonorae*	MUCL 47689	Arizona	JQ087893	JQ087920	Wu et al. (2022b)
*Fomitiporia* sp.	MUCL 53993	Mexico	JX093807	JX093851	Wu et al. (2022b)
*Fomitiporia* sp.	MUCL 53994	Mexico	JX093808	JX093852	Wu et al. (2022b)
*Fomitiporia* sp.	MUCL 53798	French Guiana	JX093811	JX093855	Wu et al. (2022b)
*Fomitiporiaspinescens*	ICN 200566	Brazil	MN918549	—	Alves-Silva et al. (2020a)
*Fomitiporiasubhippophaëicola*	Cui 12096	China	KU364421	KU364426	Chen et al. (2017)
*Fomitiporiasubhippophaëicola*	Cui 12102	China	KU364423	KU364424	Chen et al. (2017)
*Fomitiporiasubrobusta*	Dai 13576	China	KX639617	KX639635	Wu et al. (2022b)
*Fomitiporiasubrobusta*	Dai 13577	China	KX639618	KX639636	Wu et al. (2022b)
*Fomitiporiasubtropica*	Cui 9122	China	KX639622	KX639640	Wu et al. (2022b)
*Fomitiporiasubtropica*	Cui 9115	China	KX639623	KX639641	Wu et al. (2022b)
*Fomitiporiatabaquilio*	MUCL 46230	Argentina	GU461940	DQ122394	Wu et al. (2022b)
*Fomitiporiatabaquilio*	MUCL 47754	Argentina	GU461941	GU461994	Wu et al. (2022b)
*Fomitiporiatasmanica*	Dai 18793	Australia	MH971173	MH971218	Wu et al. (2022b)
*Fomitiporiatasmanica*	Dai 18799	Australia	MH971174	MH971219	Wu et al. (2022b)
*Fomitiporiatenuis*	MUCL 44802	Ethiopia	GU461957	AY618206	Amalfi et al. (2010)
*Fomitiporiatenuis*	MUCL 49948	Gabon	GU461958	GU461998	Amalfi et al. (2010)
*Fomitiporiatenuitubus*	Dai 16204	China	KX639619	KX639637	Wu et al. (2022b)
*Fomitiporiatenuitubus*	Yuan 5736	China	JQ003902	JQ003903	Wu et al. (2022b)
*Fomitiporiatexana*	MUCL 47690	USA	JQ087894	JQ087921	Amalfi et al. (2012)
*Fomitiporiatexana*	MUCL 51143	USA	JQ087895	JQ087922	Amalfi et al. (2012)
***Fomitiporiatriqueter***	**JV 2402/36**	**Ecuador**	**PV368090**	—	**Present study**
***Fomitiporiatriqueter***	**JV 2402/68**	**Ecuador**	**PV368091**	**PV368096**	**Present study**
*Fomitiporiatsitsikamensis*	CMW 47881	South Africa	MH599111	MH599121	Tchoumi et al. (2020)
*Fomitiporiatsitsikamensis*	CMW 48058	South Africa	MH599109	MH599120	Tchoumi et al. (2020)
*Fomitiporiatsitsikamensis*	CMW 48621	South Africa	MH599110	MH599123	Tchoumi et al. (2020)
*Fomitiporiatsugina*	MUCL 52702	USA	JQ087898	JQ087925	Amalfi et al. (2012)
*Fomitiporiatsugina*	MUCL 52703	USA	JQ087899	JQ087926	Amalfi et al. (2012)
*Fomitiporiauncinata*	ICN 200561	Brazil	—	MN918542	Alves-Silva et al. (2020a)
*Neophellinusuncisetus*	MUCL 46231	Argentina	GU461960	EF429235	Wu et al. (2022b)
*Neophellinusuncisetus*	MUCL 47061	Argentina	GU461972	GU462000	Wu et al. (2022b)

**Bold** = new taxa; — refers to the data unavailability.

## ﻿Results

### ﻿Molecular phylogeny

**Figure 1.**
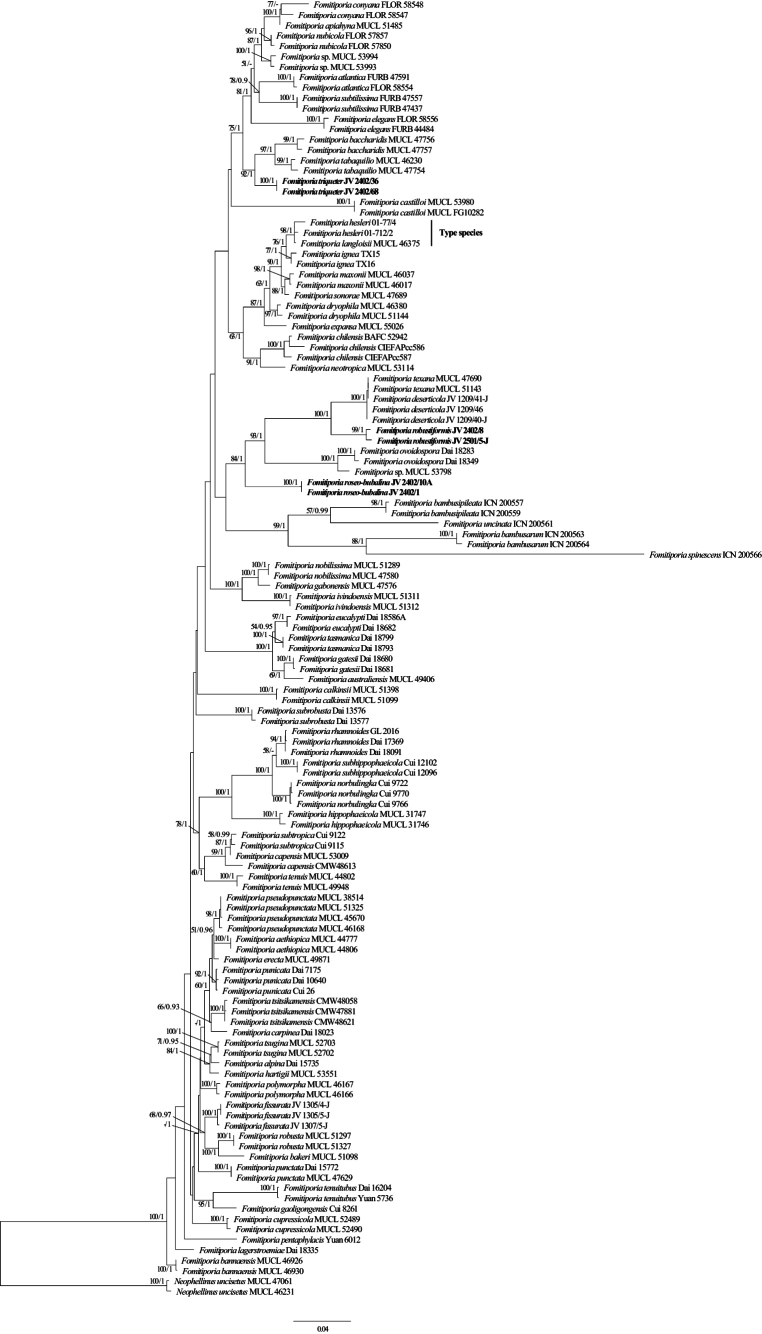
Maximum likelihood tree illustrating the phylogeny of *Fomitiporia* based on the combined dataset of ITS + nLSU sequences. The maximum likelihood bootstrap values (≥50) and Bayesian posterior probability values (≥0.90) are indicated above the branches. The new species is in bold.

The combined ITS and nLSU dataset included sequences from 123 fungal collections representing 65 taxa of *Fomitiporia*, and two samples of the genus *Neophellinus* were used as the outgroup. The final alignment comprised a total of 1,827 base pairs (bp), including 930 bp of ITS and 898 bp of nLSU. The best model was estimated and applied in the Bayesian analysis: GTR+I+G, lset nst = 6, rates = invgamma; prset statefreqpr = dirichlet (1,1,1,1). Bayesian analysis yielded a nearly congruent topology (average standard deviation of split frequencies = 0.009985) with the ML analysis; therefore, only the topology from the ML analysis is presented. The current phylogeny (Fig. 1) showed that the newly sequenced specimens fell into the *Fomitiporia* clade and formed three independent new lineages with strong support: *Fomitiporiarobustiformis* (BS = 99% in ML, BPP = 1.00), *Fomitiporiaroseo-bubalina* (BS = 100% in ML, BPP = 1.00), and *Fomitiporiatriqueter* (BS = 100% in ML, BPP = 1.00).

The ITS and nLSU sequences generated in this study were subjected to BLAST searches in the NCBI database (https://blast.ncbi.nlm.nih.gov/Blast.cgi). The BLASTN results are consistent with our phylogeny, and the top five search results for the type specimen sequences of the three new *Fomitiporia* species are provided (Tables 2, 3).

**Table 2.** BLAST results in the NCBI database with ITS.

Species	Match rank	Matched species	Identity (%)	Score	Query coverage (%)	Alignment length	GenBank accession no.	Origin
*Fomitiporiarobustiformis* (holotype: JV 2402/8)	1	*Phellinustexanus*	93.57	1123	100	783	JQ087895	USA
	2	*Phellinustexanus*	93.57	1122	100	783	JQ087894	USA
	3	*Fomitiporiadeserticola*	93.83	1110	98	744	KT381634	USA
	4	*Fomitiporiadeserticola*	93.83	1109	98	743	KT381635	USA
	5	*Phellinustexanus*	93.73	1105	98	749	KT381636	USA
*Fomitiporiaroseo-bubalina* (holotype: JV 2402/1)	1	*Fomitiporiamediterranea*	86.35	821	100	767	PQ555869	—
	2	*Fomitiporiamediterranea*	86.30	817	100	801	AY849303	Italy
	3	*Phellinuserectus*	86.30	817	100	778	GU461939	France
	4	*Fomitiporiamediterranea*	86.22	815	100	781	EF442330	—
	5	*Fomitiporiamediterranea*	86.22	815	100	766	PQ060508	Italy
*Fomitiporiatriqueter* (holotype: JV 2402/68)	1	*Fomitiporiabaccharidis*	93.38	1085	99	738	MW880690	—
	2	*Phellinustabaquilio*	92.82	1070	99	762	GU461940	Argentina
	3	*Phellinustabaquilio*	92.80	1064	99	756	GU461941	Argentina
	4	*Fomitiporiaapiahyna*	90.31	976	99	770	MN918572	Brazil
	5	*Fomitiporianubicola*	91.09	972	96	735	KU663308	Brazil

**Table 3.** BLAST results in the NCBI database with nLSU.

Species	Match rank	Matched species	Identity (%)	Score	Query coverage (%)	Alignment length	GenBank accession no.	Origin
*Fomitiporiarobustiformis* (holotype: JV 2402/8)	1	*Phellinushartigii*	97.58	1620	99	1459	PV336067	Poland
	2	*Fomitiporiatsugina*	97.58	1620	99	978	KC551838	—
	3	*Phellinushartigii*	97.58	1620	99	978	KC551856	—
	4	*Phellinusrobustus*	97.58	1620	99	1459	PV250053	Poland
	5	*Fomitiporiapunctata*	97.58	1620	99	1459	PV271020	Poland
*Fomitiporiaroseo-bubalina* (holotype: JV 2402/1)	1	*Fomitiporiapunctata*	97.87	1615	99	1459	PV271020	Poland
	2	*Fomitiporiatsugina*	97.76	1609	99	978	KC551836	—
	3	*Fomitiporiatsugina*	97.76	1609	99	978	KC551838	—
	4	*Fomitiporiapunctata*	97.76	1609	99	1459	PV277016	Poland
	5	*Phellinusrobustus*	97.76	1609	99	1459	PV250053	Poland
*Fomitiporiatriqueter* (holotype: JV 2402/68)	1	*Fomitiporiabaccharidis*	98.32	1663	100	957	MW880691	—
	2	*Fomitiporiatsugina*	98.41	1661	99	978	KC551836	—
	3	*Fomitiporiatsugina*	98.31	1657	99	978	KC551844	—
	4	*Fomitiporiatsugina*	98.31	1657	99	978	KC551839	—
	5	*Fomitiporiatsugina*	98.31	1657	99	978	KC551846	—

### ﻿Taxonomy

#### ﻿ Fomitiporia robustiformis

Taxon classificationFungiHymenochaetalesHymenochaetaceae

Jian Chen, Yuan Yuan, K.Y. Luo, Y.C. Dai & Vlasák sp. nov.

CA510350-6E11-5023-AEC8-054B770EC90E

858483

Figs 2 , 3

##### Diagnosis.

*Fomitiporiarobustiformis* is closely related to *F.texana* (Murrill) Nuss and *F.deserticola* Vlasák, but *F.texana* differs from *F.robustiformis* by the presence of hymenial setae and cystidioles, larger basidiospores (7–9 × 6.5–9 µm vs. 5–6.4 × 4.7–6.1 µm). *F.deserticola* is distinguished from *F.robustiformis* by the presence of abundant cystidioles and larger basidiospores (6–7.5 × 5.5–7 µm vs. 5–6.4 × 4.7–6.1 µm).

**Figure 2.**
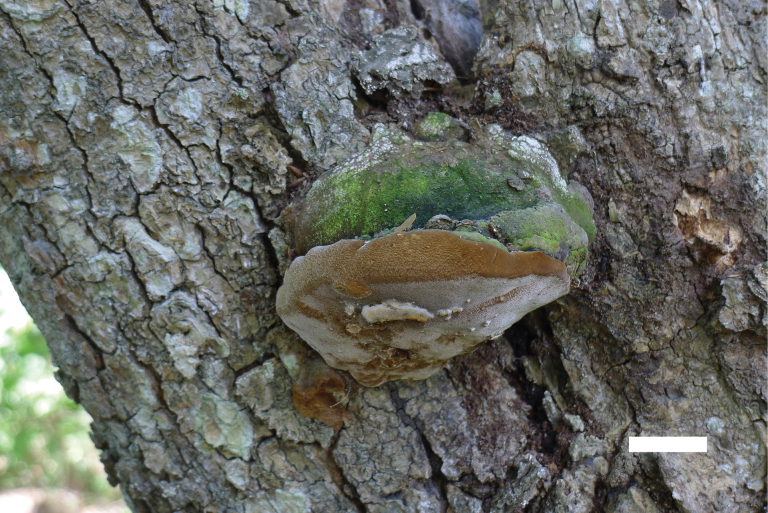
Basidiomata of *Fomitiporiarobustiformis* (holotype, JV 2402/8). Scale bar: 1 cm.

##### Holotype.

Ecuador • Arenillas, dry tropical forest, on living *Acacia* sp., 17.II.2024, leg J. Vlasák, JV 2402/8 (BJFC 053711).

**Figure 3.**
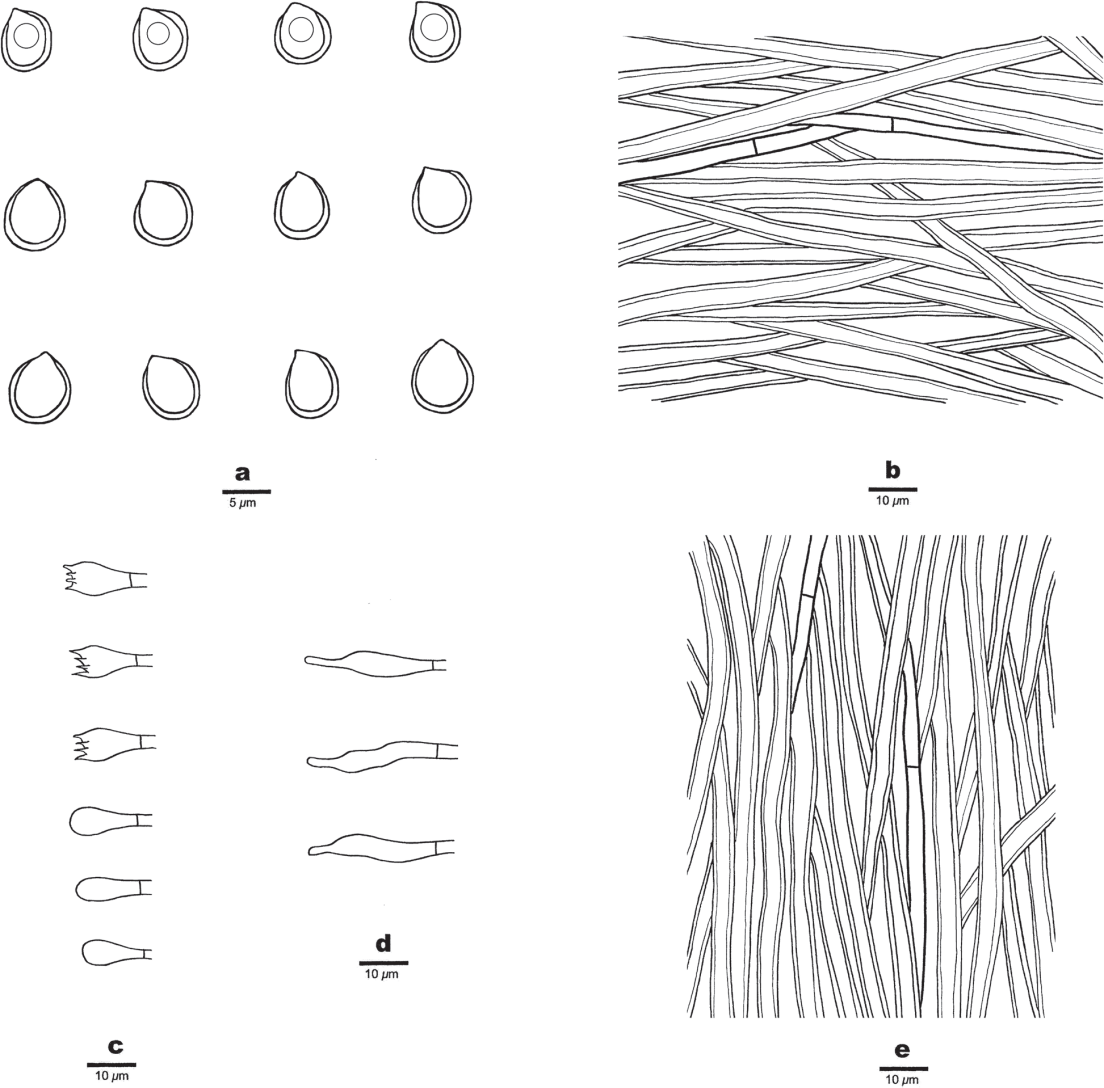
Microscopic structures of *Fomitiporiarobustiformis* (Holotype, JV 2402/8) **a** basidiospores **b** hyphae from context **c** basidia and basidioles **d** cystidioles **e** hyphae from the tube trama.

##### Etymology.

*Robustiformis* (Lat.) refers to a new species characterized by robust basidiomata.

##### Description.

Basidiomata perennial, pileate, inseparable, sessile, without distinctive odor or taste when fresh, woody hard in consistency, light in weight when dry; pilei ungulate, fan-shaped, projecting up to 5 cm, 5 cm wide, and 3 cm thick at base; pileal surface brown, sometimes greenish from algae, glabrous; margin broad and rounded. Pore surface pale mouse-gray when fresh, becoming yellowish brown when dry, not glossy; sterile margin brown, up to 1 mm wide; pores circular to angular, 6–7 per mm; dissepiments thin, entire. Context yellow-brown, woody hard, up to 7 mm thick; tubes pale gray to yellow-brown, paler than pore surface, woody hard, up to 2 cm long, annual layers indistinct.

***Hyphal structure*.** Hyphal system dimitic, generative hyphae simple septate; all hyphae IKI−, CB−; tissue becoming dark brown in KOH.

***Context*.** Generative hyphae frequent, pale yellow, slightly thick-walled, rarely branched, frequently septate, 3–3.5 µm in diam; skeletal hyphae dominant, yellowish brown, thick-walled, unbranched, more or less flexuous, interwoven, 3–4.5 µm in diam.

***Tubes*.** Generative hyphae pale yellowish, slightly thick-walled, rarely branched, frequently septate, 3–3.5 µm in diam; skeletal hyphae dominant, yellowish, thick-walled, unbranched, more or less straight, parallel along the tubes, 3–4.5 µm in diam. Hymenial setae absent; cystidioles present, fusoid, hyaline, thin-walled, 14–18 × 2.5–3.5 μm; basidia clavate, with four sterigmata and a simple septum at the base, 10–18 × 6–7.5 µm; basidioles dominant in hymenium, in shape similar to basidia, but slightly smaller.

Basidiospores globose, hyaline, thick-walled, smooth, some with a guttule, IKI[+], CB+, (4.4−)5–6.4(−7.1) × (4.4−)4.7–6.1(−6.9) µm, L = 5.66 µm, W = 5.39 µm, Q = 1.05 (n = 30/1).

***Type of rot*.** White rot.

##### Additional specimen (paratype) examined.

Peru • Chongoyape, dry tropical forest, on a dead branch of a living angiosperm tree, 21.I.2025, leg J. Vlasák Jr., JV 2501/5-J.

#### ﻿ Fomitiporia roseo-bubalina

Taxon classificationFungiHymenochaetalesHymenochaetaceae

Jian Chen, Yuan Yuan, K.Y. Luo, Y.C. Dai & Vlasák sp. nov.

4041AC3B-32B8-545C-A165-91DC7610192A

858484

Figs 4 , 5

##### Diagnosis.

*Fomitiporiaroseo-bubalina* is related to *F.ovoidospora* Y.C. Dai & F. Wu, but *F.ovoidospora* differs from *F.roseo-bubalina* by perennial basidiocarps, the presence of fusoid cystidioles, smaller pores (9–10 per mm vs. 4–5 per mm), and smaller basidiospores (4.7–5.5 × 3.8–5 µm vs. 5.3–6.7 × 5–6.2 µm).

**Figure 4.**
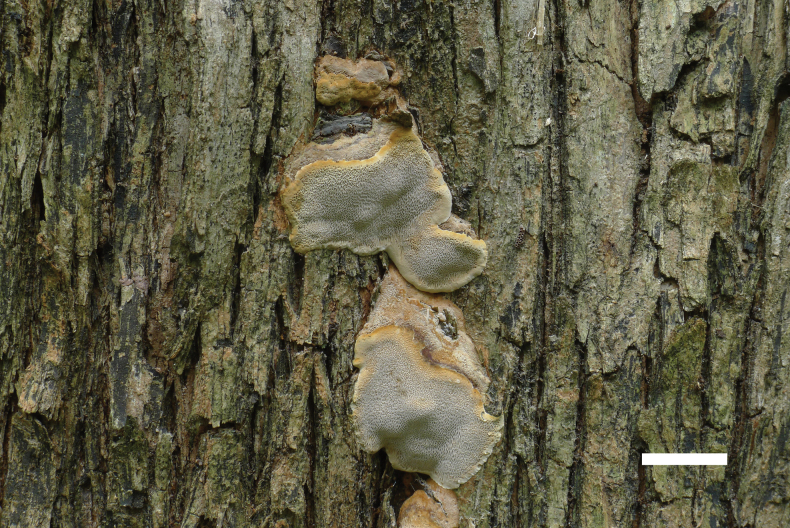
Basidiomata of *Fomitiporiaroseo-bubalina* (Holotype, JV 2402/1). Scale bar: 1 cm.

##### Holotype.

Ecuador • Arenillas, dry tropical forest, on the base of living *Acacia*, 17.II.2024, leg J. Vlasák, JV 2402/1 (BJFC 053710).

**Figure 5.**
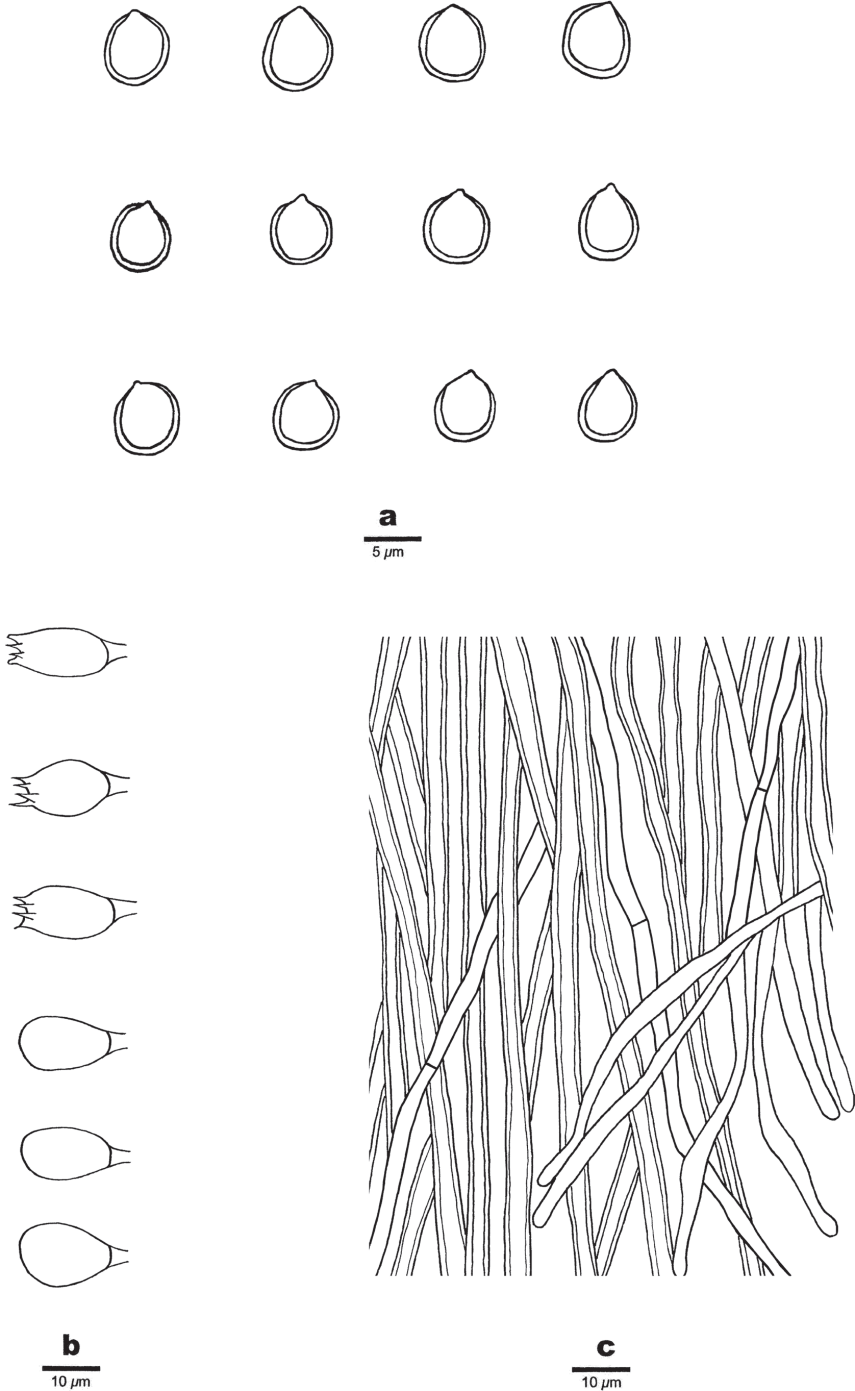
Microscopic structures of *Fomitiporiaroseo-bubalina* (Holotype, JV 2402/1) **a** basidiospores **b** basidia and basidioles **c** hyphae from the tube trama.

##### Etymology.

*Roseo-bubalina* (Lat.) refers to the new species characterized by pink-buff pores when dry.

##### Description.

Basidiomata annual to perennial, resupinate to pileate, inseparable, growing around bark fissures at the base of living trees in shelf-like, vertical assemblages of triquetrous pilei, without distinctive odor or taste when fresh, woody hard in consistency; pilei slightly ungulate, projecting up to 2.5 cm, 3 cm wide, and 0.5 cm thick at base; pileal surface grayish, glabrous; margin blunt. Pore surface pink buff when dry, glossy; sterile margin cream, up to 1 mm wide; pores circular to angular, 4–5 per mm; dissepiments thin, entire. Context very thin; tubes yellowish brown, paler than pore surface, woody hard, up to 2 mm long, annual layers indistinct.

***Hyphal structure*.** Hyphal system dimitic, generative hyphae simple septate; all hyphae IKI−, CB−; tissue becoming dark brown in KOH.

***Tubes*.** Generative hyphae pale yellowish, slightly thick-walled, rarely branched, frequently septate, 2.5–3.5 µm in diam; skeletal hyphae dominant, yellowish, thick-walled, unbranched, more or less straight, parallel along the tubes, 3.5–4.8 µm in diam. Hymenial setae absent; cystidia and cystidioles absent; basidia subglobose to clavate, with four sterigmata and a simple septum at the base, 10–18 × 6–9 µm; basidioles dominant in hymenium, in shape similar to basidia, but slightly smaller.

Basidiospores globose, hyaline, thick-walled, smooth, IKI[+], CB+, (5−)5.3–6.7(−7.4) × (4.9−)5–6.2(−6.6) µm, L = 6.01 µm, W = 5.60 µm, Q = 1.07 (n = 30/1).

***Type of rot*.** White rot.

##### Additional specimen (paratype) examined.

Ecuador • Arenillas, dry tropical forest, on the base of living *Acacia*, 17.II.2024, leg J. Vlasák, JV 2402/10A.

#### ﻿ Fomitiporia triqueter

Taxon classificationFungiHymenochaetalesHymenochaetaceae

Jian Chen, Yuan Yuan, K.Y. Luo, Y.C. Dai & Vlasák sp. nov.

44F2B9A5-73C7-5C2B-8749-9C5B57472833

858485

Figs 6 , 7

##### Diagnosis.

*Fomitiporiatriqueter* is closely related to *F.baccharidis* and *F.tabaquilio* (Urcelay, Robledo & Rajchenb.) Decock & Robledo, and they all occur in South America. However, the latter two species differ from *F.triqueter* in having larger basidiospores (5.5–6.5 × 4.8–5.8 μm in *F.baccharidis*, 6–7.5 × 8–6.5 μm in *F.tabaquilio*, vs. 3.4–5.5 × 3.2–5.4 μm in *F.triqueter*).

**Figure 6.**
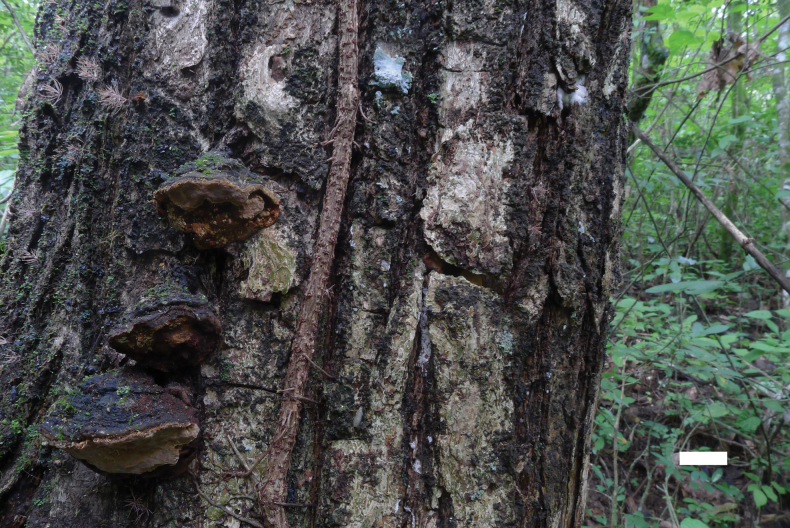
Basidiomata of *Fomitiporiatriqueter* (holotype, JV 2402/68). Scale bar: 1 cm.

##### Holotype.

Ecuador • Macará, dry tropical forest, on the thick bark of a living angiosperm tree, 23.II.2024, leg J. Vlasák Jr., JV 2402/68 (BJFC 053713).

**Figure 7.**
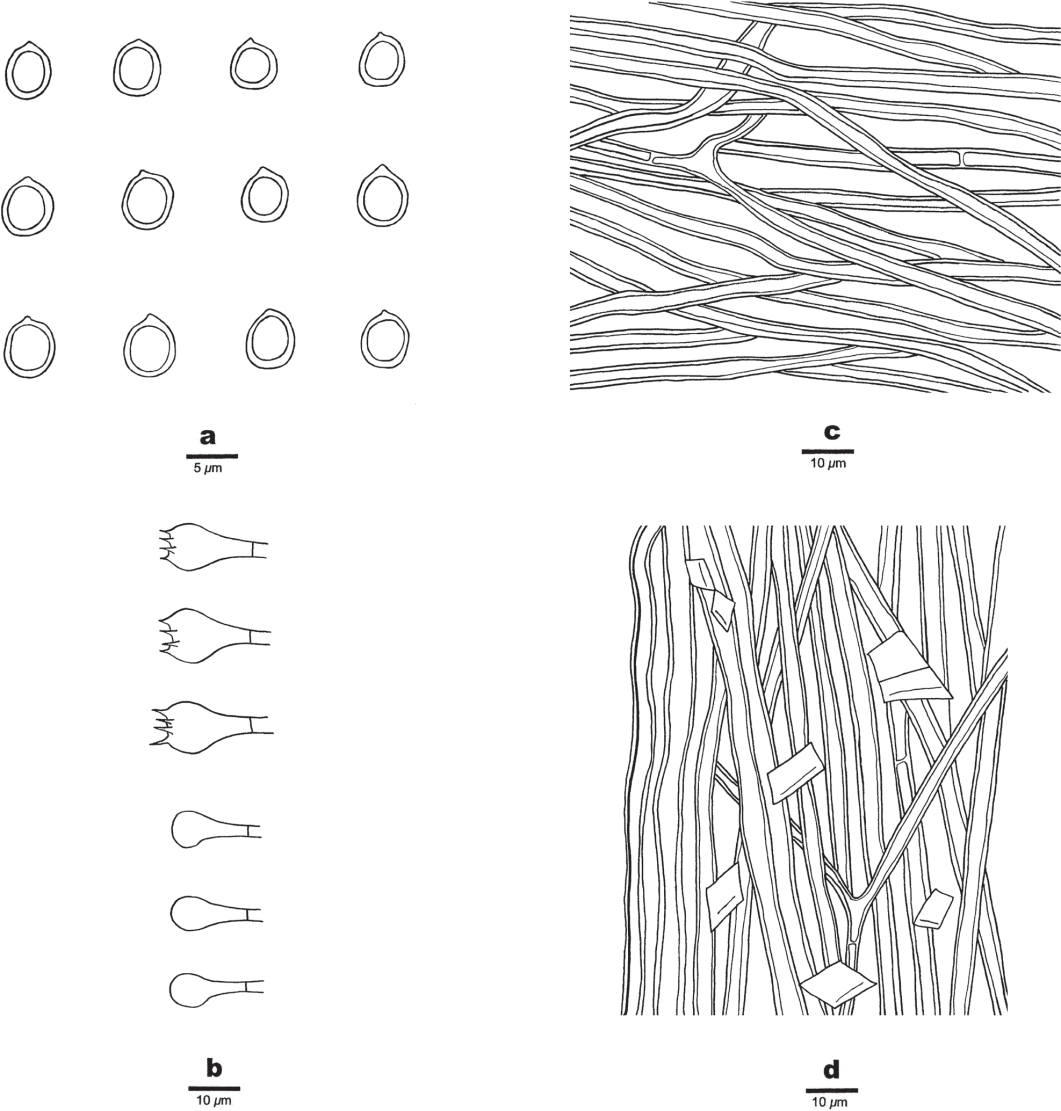
Microscopic structures of *Fomitiporiatriqueter* (holotype, JV 2402/68) **a** basidiospores **b** basidia and basidioles **c** hyphae from context **d** hyphae from the tube trama.

##### Etymology.

*Triqueter* (Lat.) refers to the new species characterized by triqueter basidiomata in section.

##### Description.

Basidiomata biennial to perennial, but evidently short-living, pileate, without distinctive odor or taste when fresh, woody hard in consistency; pilei ungulate, triquetrous in section, projecting up to 4 cm, 2.5 cm wide, and 2 cm thick at base; pileal surface grayish dark, concentrically sulcate, glabrous, soon cracked and blackish with age; margin sharp; pore surface yellowish brown when fresh, becoming brown when dry; sterile margin yellowish brown, up to 100 µm wide; pores circular, 9–10 per mm; dissepiments slightly thick, entire. Context yellowish brown, woody hard, up to 2 mm thick; tubes yellowish brown, paler than pore surface, woody hard, up to 2 cm long, annual layers indistinct.

***Hyphal structure*.** Hyphal system dimitic, generative hyphae simple septate; all hyphae IKI−, CB−; tissue becoming dark brown in KOH.

***Context*.** Generative hyphae frequent, pale yellow, slightly thick-walled, occasionally branched, frequently septate, 2.5–3 µm in diam; skeletal hyphae dominant, yellowish brown, thick-walled, unbranched, more or less flexuous, interwoven, 3–4 µm in diam.

***Tubes*.** Generative hyphae golden, slightly thick-walled, occasionally branched, frequently septate, 2.5–3.5 µm in diam; skeletal hyphae dominant, yellowish, thick-walled with a medium lumen, unbranched, more or less straight, parallel along the tubes, 3–4.5 µm in diam. Hymenial setae absent; cystidia and cystidioles absent; basidia subglobose to capitate, with four sterigmata and a simple septum at the base, 13–18 × 7–11 µm; basidioles dominant in hymenium, in shape similar to basidia, but slightly smaller; large rhomboid crystals present in the hymenium.

Basidiospores globose, hyaline, thick-walled, smooth, IKI[+], slightly CB+, (3.1−)3.4–5.5(−5.7) × (2.9−)3.2–5.4(−5.8) µm, L = 4.57 µm, W = 4.39 µm, Q = 1.04–1.05 (n = 60/2).

***Type of rot*.** White rot in the bark; no signs of wood rot under the infested bark.

##### Additional specimen (paratype) examined.

Ecuador • Macará, dry tropical forest, on the thick bark of a living angiosperm tree, 21.II.2024, leg J. Vlasák Jr., JV 2402/36 (BJFC 053712).

## ﻿Discussion

Four species of *Fomitiporia* from Ecuador have been reported in the last 10 years, including *F.baccharidis* (Pat.) Decock et al., *F.conyana* Alves-Silva & Drechsler-Santos, *F.nubicola* Alves-Silva et al., and *F.impercepta* Morera et al. (Wu et al. 2022b). In the present study, three new species of *Fomitiporia* are described, all of which share the typical characteristics of *Fomitiporia*, including subglobose, hyaline, thick-walled, smooth, dextrinoid, and cyanophilous basidiospores. The phylogenetic analysis revealed that they are distantly related to other taxa in the genus (Fig. 1).

Phylogenetically, *Fomitiporiarobustiformis* is closely related to *F.texana* (Murrill) Nuss and *F.deserticola* Vlasák (Fig. 1), but *F.texana* differs from *F.robustiformis* by the presence of hymenial setae and cystidioles, larger pores (4–6 per mm vs. 6–7 per mm), larger subglobose basidiospores (7–9 × 6.5–9 µm vs. 5–6.4 × 4.7–6.1 µm), and occurrence in the Southwest USA (Larsen and Cobb-Poulle 1990). *F.deserticola* is distinguished from *F.robustiformis* by the presence of rare hymenial setae and abundant cystidioles, larger pores (4–6 per mm vs. 6–7 per mm), larger and subglobose basidiospores (6–7.5 × 5.5–7 µm vs. 5–6.4 × 4.7–6.1 µm), and a distribution in Arizona, USA (Vlasák and Vlasák 2016). Morphologically, *F.robustiformis* is most similar to *F.subrobusta* B.K. Cui & Hong Chen by sharing perennial, pileate basidiocarps; approximately the same size pores; the presence of cystidioles; and the absence of hymenial setae. However, *F.subrobusta* has larger and subglobose to obovoid basidiospores (6.2–6.8 × 5.2–6 μm), and its distribution is in South China (Chen and Cui 2017).

Morphologically, *Fomitiporiaroseo-bubalina* is characterized by annual to perennial basidiocarps, absent hymenial setae, and globose basidiospores, which is similar to *F.pentaphylacis* L.W. Zhou (Zhou and Xue 2012). However, *F.pentaphylacis* differs from *F.roseo-bubalina* by its smaller pores (6–9 per mm vs. 4–5 per mm), larger basidiospores (5.9–7.6 × 5.4–6.5 µm vs. 5.3–6.7 × 5–6.2 µm), the presence of cystidioles, and its distribution in South China. Phylogenetically, *F.roseo-bubalina* is related to *F.ovoidospora* Y.C. Dai & F. Wu, but *F.ovoidospora* differs from *F.roseo-bubalina* by perennial basidiocarps, dimidiate or triquetrous and cracked pilei, the presence of fusoid cystidioles, smaller pores (9–10 per mm vs. 4–5 per mm), and smaller and ovoid basidiospores (4.7–5.5 × 3.8–5 µm vs. 5.3–6.7 × 5–6.2 µm). Furthermore, *F.ovoidospora* grows on *Khaya* or other angiosperm wood and has a distribution in Vietnam.

*Fomitiporiatriqueter* is characterized by biennial to perennial and pileate basidiomata, ungulate to triquetrous and cracked pileal, the absence of hymenial setae and cystidioles, the presence of large rhomboid crystals in the hymenium, and small and globose basidiospores (3.4–5.5 × 3.2–5.4 μm). *F.triqueter* and *F.ovoidospora* share similar pores and basidiospores, but the latter has cystidioles and lacks rhomboid crystals (Wu et al. 2022b). Phylogenetically, *F.triqueter* is closely related to *F.baccharidis* and *F.tabaquilio* (Urcelay, Robledo & Rajchenb) Decock & Robledo, and they all occur in South America. However, *F.baccharidis* is different from *F.triqueter* by its velutinate and uncracked pileal, strongly glossy pore surface, and larger basidiospores (5.5–6.5 × 4.8–5.8 μm vs. 3.4–5.5 × 3.2–5.4 µm, Amalfi et al. 2014). *Fomitiporiatabaquilio* differs from *F.triqueter* by its glossy pore surface, the presence of cystidioles, larger and subglobose basidiospores (6–7.5 × 8–6.5 µm vs. 3.4–5.5 × 3.2–5.4 µm), and its distribution in Argentina (Decock et al. 2007).

## Supplementary Material

XML Treatment for Fomitiporia robustiformis

XML Treatment for Fomitiporia roseo-bubalina

XML Treatment for Fomitiporia triqueter

## ﻿Additional information

### Author ORCIDs

Jian Chen https://orcid.org/0000-0001-8409-7791

Josef Vlasák https://orcid.org/0000-0002-5363-4459

Kai-Yue Luo https://orcid.org/0000-0001-6145-4864

Yuan Yuan https://orcid.org/0000-0001-6674-9848

### Data availability

All of the data that support the findings of this study are available in the main text.
